# Effect of psychological interventions on outcomes for caregivers of hematopoietic stem cell transplant patients: Protocol for a systematic review and planned meta-analysis

**DOI:** 10.1371/journal.pone.0330323

**Published:** 2025-08-18

**Authors:** Ranjita Poudel, Min-Jeong Yang, Skye O. Dougan, Helen Yates, Bradley Brown, Sierra Washington, Aarya P. Satardekar, Mary Katherine Haver, Christine Vinci

**Affiliations:** 1 Department of Health Outcomes and Behavior, Moffitt Cancer Center, Tampa, Florida, United States of America; 2 Institute for Nicotine and Tobacco Studies, Rutgers Biomedical and Health Sciences (Rutgers Health), Rutgers, The State University of New Jersey, New Brunswick, New Jersey, United States of America; 3 Department of Health Behavior, Society, and Policy, School of Public Health, Rutgers, The State University of New Jersey, Piscataway, New Jersey, United States of America; 4 Department of Psychology, University of South Florida, Tampa, Florida, United States of America; 5 Samuel P Bell III College of Public Health, University of South Florida, Tampa, Florida, United States of America; 6 Biomedical Library, Moffitt Cancer Center, Tampa, Florida, United States of America; 7 Department of Oncological Sciences, University of South Florida, Tampa, Florida, United States of America; Azienda Ospedaliera Universitaria SS Antonio e Biagio e Cesare Arrigo, Alessandria, University of Eastern Pedemont, ITALY

## Abstract

Caregivers of Hematopoietic Stem Cell Transplant (HSCT) recipients bear significant responsibilities involving commitment to be available 24/7, up to at least 100 days post-transplant or longer, and to provide emotional and physical support to the recipient. This extended caregiving, along with managing all other household responsibilities, can result in an increased caregiver burden, which is associated with reduced quality of life (QoL) and compromised psychological, and physical well-being. In recent years, there has been increased attention to interventions addressing the mental health outcomes of caregivers of HSCT patients. However, there remains a limited understanding of the feasibility and effectiveness of existing interventions targeted to this population. This review aims to systematically evaluate existing psychological interventions for caregivers of HSCT patients and report on: 1) the feasibility and acceptability of these interventions, and 2) the efficacy of these interventions on caregivers’ psychological outcomes. A systematic literature search for studies evaluating psychological interventions targeted to caregivers of HSCT patients was conducted in English through the following databases: Ovid MEDLINE (Wolters Kluwer), Embase (Elsevier), CINAHL Complete (EBSCO), APA PsycArticles (EBSCO), APA PsycInfo (EBSCO). Two reviewers will independently identify the studies, extract relevant data, and conduct quality and risk of bias assessments. Discrepancies in coding will be discussed between the two reviewers, and a third reviewer will resolve pending disagreements. Within the systematic review, all eligible studies will be summarized descriptively. If data allows, we will further utilize meta-analysis to quantitatively evaluate the efficacy of these psychological interventions tested in RCTs. The association between intervention type, modality, duration, and efficacy will be assessed via subgroup analysis. Ethics approval is not required as this study will only include secondary data from already published studies. The outcomes will be shared through scientific conferences and peer-reviewed publications. This protocol is registered in PROSPERO: CRD42024489695.

## Introduction

Hematopoietic Stem Cell Transplant (HSCT) is a widely used treatment for various hematological malignancies [1]. This treatment is very intensive, requiring a dedicated caregiver, often a family member or a friend of the recipient, for an extended period [2,3]. Caregivers of HSCT recipients bear significant responsibilities involving a commitment to be available 24/7, up to 100 days post-transplant or longer if there are any complications, to provide emotional and physical support to the recipients [4]. This intense caregiving, along with managing all the household responsibilities, can result in an increased caregiver burden which is associated with reduced quality of life (QoL) and compromised psychological, and physical well-being among caregivers which further impacts patient outcomes [5,6].

Existing data indicate elevated levels of burden, distress, anxiety, and depression among the caregivers of HSCT patients [7,8]. For instance, a comprehensive review examining the caregiving experience among caregivers of HSCT patients showed that they displayed distress levels comparable to or even higher than the patient’s reported distress level [9]. Furthermore, emerging literature reports the impact of caregiving stress on metabolic, inflammatory, and immunological markers among the caregivers of HSCT patients, potentially leading to a range of health consequences [10–12]. The compromised well-being among caregivers may translate to inefficient patient support, subsequently impacting patient outcomes (e.g., anxiety, depression, healthcare utilization) [13]. These findings underscore the need for psychological interventions to address caregiving stress in this population and to improve the well-being of both caregivers and patients.

In response to the pressing need for psychological interventions among the caregiving population, a body of literature on interventions focused on coping, self-care, stress management, and well-being among caregivers of HSCT patients is growing [6,14–16]. These interventions teach strategies to cope with caregiving stress (e.g., stress reappraisal) to help reduce emotional difficulties among caregivers and improve outcomes for both caregivers and patients. Given the heterogeneous study designs of the existing studies (e.g., feasibility, cross-sectional studies, cohort studies, randomized controlled trials [RCTs]), synthesizing the findings could identify commonalities and gaps in the literature to inform future intervention development and evaluation. Towards this goal, our systematic review will examine whether existing psychological interventions for caregivers of HSCT patients are feasible, acceptable, and efficacious for improving caregiver’s psychological outcomes. Feasibility (e.g., recruitment, retention, and adherence) and acceptability (e.g., acceptability measures depending on the study) metrics provide evidence that help determine whether an intervention should be recommended for larger-scale testing [17]. Given the field is in an early stage, synthesizing the literature regarding the feasibility and acceptability of existing interventions will guide the next steps for future research. This review will expand on the findings from the only existing systematic review on psychological interventions for caregivers of HSCT patients, published in 2018, that reported outcomes from 12 studies, including 4 RCTs [5]. Findings will serve as a comprehensive summary of up-to-date, evidence-based psychological interventions for caregivers of HSCT patients, building on previous work [5] with an additional meta-analysis component.

Our systematic review on psychological interventions for caregivers of HSCT patients aims to narratively report on: 1) the feasibility and acceptability of existing psychological interventions for caregivers of HSCT patients, and 2) the efficacy of these interventions on caregivers’ psychological outcomes (e.g., burden, distress, anxiety, depression). If data allows, we will further utilize meta-analysis to quantitatively address the following questions: 1) Do psychological interventions for caregivers of HSCT patients significantly reduce psychological symptoms at the end of treatment and/or follow-up compared to a control condition? and 2) Are the type, modality, and duration of the intervention associated with treatment efficacy at the end of treatment and/or follow-up?

## Methods

The development of this protocol adhered to the guidelines of Preferred Reporting Items for Systematic Review and Meta-Analysis Protocols (PRISMA-P) [18]. See supporting information (S1 Checklist) for PRISMA-P checklist. We registered this protocol (CRD42024489695) using the Prospective Register of Systematic Reviews Protocols (PROSPERO). Any amendments to the review protocol will be reported within this register.

### Eligibility criteria

This systematic review will only include quantitative studies assessing psychological outcomes for caregivers of HSCT patients (e.g., burden, distress, anxiety, depression). The eligibility criteria include: 1) quantitative studies in peer-reviewed journals; 2) with and without control conditions (e.g., one-arm studies, feasibility studies), 3) available in English language unrestricted by the geographic location, 4) regardless of the publication date, 5) focusing on adult caregivers (≥18 years) of either the allogeneic or autologous HSCT patients, and 6) evaluating a psychological intervention. For the meta-analysis, additional inclusion criteria will be: 1) RCTs including an intervention condition and control condition(s) and 2) intervention effect on psychological outcomes assessed at least one-time post-baseline. Exclusion criteria for both the systematic review and meta-analysis include: 1) studies involving formal caregivers (who provide caregiving as a paid service), 2) studies evaluating interventions that are not consistent with traditional psychological interventions (e.g., interventions that deliver only music or physical activity).

Outcomes of interest are caregivers’ psychological outcomes (e.g., burden, distress, depression, and anxiety). If available, additional physiological and biological measures of stress (e.g., endocrine, metabolic, and immunological outcomes) and patient-reported outcomes (e.g., anxiety, depression, healthcare utilization) will be examined.

### Information sources and search strategy

The following electronic bibliographic databases were searched: Ovid MEDLINE® ALL (Wolters Kluwer); Embase (Elsevier); CINAHL Complete (EBSCO); APA PsycArticles (EBSCO); APA PsycInfo (EBSCO). The search strategy comprises Medical subject headings [MeSH], database thesaurus, keywords, phrase terms relating to caregivers, hematopoietic stem cell transplant, and psychological interventions. No search hedges, filters, or limits were applied within the search strategies. The review team evaluated each study based on inclusion and exclusion criteria during title abstract and full-text review. If a study was published in non-English language, the review team checked for a reliable English translated version. If one was not available, the team excluded the study due to a lack of medical-level translation services for reliable translation. Initial database search strategies were run on February 19, 2024. The Ovid MEDLINE ® ALL 1946 to February 16, 2024, search strategy is provided in supporting information (S2 Text). Most recent search utilizing the same strategy was conducted in February 2025. Results were imported into EndNote and exported via a.xml file into Covidence. Upon completion of Full-text screening, the research team scanned the reference lists of the included studies as well as excluded review/meta-analysis papers to identify any additional relevant publications not obtained through the search iterations. Currently, abstract and full-text screening has been completed, and the data extraction is in progress. Results are expected to be generated by September 2025.

### Data management and screening process

Search results will be imported into EndNote 20 (Clarivate), a citation management system. A database Group Set is created within the library, and results are added to the date-specific Group. Results will be exported via a.xml file to be imported into Covidence, a web-based collaboration software platform that streamlines the production of systematic and other literature reviews. Commencing with the Ovid MEDLINE.xml file, each subsequent results.xml file will be imported, and the number of duplicate records will be noted and removed. The results will be imported into the Title and Abstract screening section.

Two reviewers will independently scan titles and abstracts to determine relevancy based on the inclusion/exclusion criteria during this phase (Phase I). Any conflicts or records with one “yes” and one “no” vote will be evaluated and resolved by a third reviewer, an arbitrator. Records receiving “yes” votes by the reviewers or reviewer/arbitrator will move forward to Phase II (i.e., full-text review). Identical steps, as noted here for Phase I, will be used for Phase II, Phase III (data extraction), and screening of the RCTs for meta-analysis. For records requiring clarification of eligibility to reach a consensus, the review team will make three attempts to obtain the necessary information from the lead author. In accordance with the PRISMA checklist, all excluded studies will be documented within their Covidence record, and notes will be available for viewing via the PRISMA flow diagram.

### Data extraction and synthesis

Phase III of the systematic review will involve data extraction. We will use a data extraction form in Covidence tailored to our specific data items. Prior to initiating data extraction, a calibration exercise will be conducted to pilot and refine the data items. This exercise will ensure consistency across all the reviewers involved. Additionally, the reviewers will also screen the studies included within the systematic review against the eligibility criteria for the meta-analysis.

The design of the data extraction template for this study will be informed by variables used by existing publication [5] as well as the recommendations from the Cochrane Handbook for Systematic Reviews of Interventions [19]. The following information will be extracted: details on publication (e.g., author names, date of publication, journal, title), study characteristics (e.g., study design, participants/population, relationship to the patients, methodology), psychological intervention (e.g., type, modality, duration), feasibility (e.g., recruitment, retention, adherence), acceptability (e.g., measures of intervention acceptability), and psychological outcomes (e.g., burden, distress, anxiety, depression). Regarding the meta-analysis, we will extract quantitative data from RCTs, including but not limited to psychological outcomes of the caregivers at the end of treatment as well as any follow-ups and the sample sizes for both the intervention condition and the control condition (see supporting information S3 Table for further details on data to be extracted). In case of missing results and/or unreported data, we will contact the original authors to obtain the data to avoid the risk of bias in the meta-analysis. We will also contact the authors via email to clarify uncertainties about the extracted information. In both cases, a maximum of three attempts will be made to contact the authors.

Overall, studies meeting inclusion criteria will first be summarized descriptively in a narrative synthesis. The synthesis will be organized by participant characteristics, study design, sample size, primary intervention type, modality, comparison group characteristics, feasibility and acceptability of the intervention, and psychological outcomes. Additionally, a descriptive summary table will be presented. The meta-analysis will involve quantitative analysis of the data from eligible RCTs.

### Assessment of methodological quality

#### Quality and risk of bias assessment.

The Quality Assessment Tool for quantitative studies [20], as recommended by the Cochrane Handbook for Systematic Reviews of Interventions [19], will be used to assess the quality of all the studies. The findings from the quality assessment will be reported in a table.

For the RCTs, we will use Cochrane Risk of Bias Tool 5.0.2 to assess for possible risk of bias [21,22]. Individual trials will be assessed for risk of bias based on five domains: randomization process, intervention deviation, missing outcome data, outcome measurement, and selective reporting. Ratings for each domain will include ‘low risk’, ‘high risk,’ or ‘some concern’. The results will be visualized in a figure for individual studies, ‘risk of bias summary’, and ‘risk of bias graph’ for all studies.

Two reviewers will complete quality assessments and risk of bias assessments. Any disagreement in coding will be resolved via discussion among these reviewers, and a third reviewer will resolve any pending discrepancies. In case of a lack of detailed information required to make a decisive judgment, an individual study will get an ‘unclear’ rating, and three subsequent attempts will be made to contact the study authors for clarification.

### Data analyses

#### Measure of treatment effect within the RCTs (meta-analysis).

Data from psychological outcomes (e.g., burden, distress, anxiety, depression) and the sample sizes from the eligible RCTs will be used to compute the intervention effect sizes and standard errors for the meta-analysis. We will compute Hedge’s g as a measure of effect size (i.e., standardized weighted mean differences for continuous measures, correcting for small sample size bias), which will be calculated using mean gain scores (i.e., pre-post) change for intervention and control conditions. This effect size represents the degree to which the intervention group changed compared to the control group in standard deviation units. The positive effect sizes will indicate greater improvement in the intervention compared to the control. Effect sizes will be considered as minimal (g < 0.20), small (0.20 ≤ g < 0.50), moderate (0.50 ≤ g < 0.80), and large (g ≥ 0.80) [23] and will be considered statistically significant with *p *< 0.05.

### Unit of analysis

The meta-analysis will only include RCTs. For RCTs examining multiple treatment conditions (e.g., active control condition), treatment condition-specific data will be extracted for each relevant study (e.g., sample size within intervention and control groups).

### Heterogeneity

The degree of heterogeneity will be computed using *I*^2^ statistics and Cochrane’s Q homogeneity test. Following are the degrees of indication of heterogeneity: 0%–25% indicating might not be important; > 25%– 50% indicating moderate heterogeneity; > 50%–75% indicating substantial heterogeneity; > 75% indicating high, considerable heterogeneity [24,25]. Potential moderators will be examined if significant heterogeneity is present [24]. Effects of potential moderators (e.g., caregiver age, gender, race) with adequate data will be examined using meta-regression analyses with restricted maximum likelihood models. Each moderator will be examined independently to maximize the number of studies included in the analyses.

### Quantitative data synthesis

According to the guidelines provided by the Cochrane Handbook for Systematic Reviews of Interventions [19], we will use RevMan V.5.4 Software for Quantitative data synthesis. In terms of between-trial heterogeneity, an overall summary intervention effect estimate will be calculated using a random-effects model across all eligible RCTs. In case of high inter-study heterogeneity (I^2 ^> 75%), a forest plot for individual studies with the narrative summary will be reported [26].

### Subgroup and sensitivity analysis

If substantial heterogeneity is observed among the RCTs (I^2^ ≥ 50% or *p *< 0.05), the source of heterogeneity will be identified by conducting subgroup analyses. This will depend on the adequacy of data to conduct subgroup analyses to explore the sources of heterogeneity in terms of certain characteristics (e.g., treatment type for patient [autologous and allogeneic], intervention type [e.g., Cognitive behavioral therapy, mindfulness meditation], and modality [e.g., digital, in-person]). Further, a sensitivity analysis will be used to examine the effect of including trials with high attrition rates or missing data on overall treatment effects from an available case analysis.

### Assessing confidence in evidence

#### Publication and outcome reporting bias.

We will assess publication bias to ensure the validity and generalization of the meta-analysis result if ten or more RCTs are eligible. Publication bias will be identified using various methods including funnel plot asymmetry, and correlation-based tests (e.g., Begg’s rank test and Egger’s regression test) [27,28]. If publication bias is identified, p-curve analysis will be conducted to calculate the true effect size [29]. Further, to assess the outcome reporting bias, we will utilize the revised Cochrane Risk of Bias Tool V.5.0.2 (RoB 2) to evaluate the risk of bias assessment [22].

### Strength of the evidence

The Grading of Recommendations Assessment, Development, and Evaluation (GRADE) approach will be used to assess the strength of the body of evidence [30]. The quality of the evidence will be checked for individual sections of the framework: risk of bias, imprecision, inconsistency, indirectness, and publication bias. Each domain will further be rated for Certainty as very low, low, moderate, or high based on the GRADE approach including RCT in the meta-analysis.

### Patient and public involvement

No patient or public participation will occur in the design, conduct, reporting, or dissemination of the systematic review/meta-analysis.

## Discussion

This protocol aims to describe the method for a systematic review and a planned meta-analysis of studies including psychological interventions addressing the needs of caregivers of HSCT patients. Given the most recent review related to psychological interventions focused on caregivers of HSCT patients was published in 2018, an updated review can advance this line of research and identify areas for future directions. Building on the methodologies employed in the previous paper, we will conduct a systematic review of psychological interventions with an additional meta-analysis component among RCTs for a more robust assessment of intervention efficacy. We will further assess the methodological quality of the existing studies using the GRADE system for comprehensive evaluation of evidence across multiple study designs.

Findings will identify emerging trends in caregiver psychological interventions (e.g., intervention type, technology-enabled delivery methods, novel interventions), providing valuable insights that inform future intervention development. Feasibility, acceptability, and efficacy outcomes from existing studies that address the needs of HSCT caregivers will be reported, identifying intervention characteristics that are most accessible and effective. Further, results will identify existing gaps in the literature (e.g., underrepresentation of interventions addressing certain populations, such as adolescents and young adults [AYAs], need for methodological refinement, and use of technology). Understanding these gaps will drive future research in this area, including avenues for implementing and disseminating psychological interventions among HSCT caregivers. This study will assess confidence in the evidence by reporting biases as well as strengths of the cumulative findings related to the feasibility, acceptability, and intervention efficacy for psychological outcomes. With systematic quality assessment, this review will evaluate the rigor, credibility, and validity of the included studies to ensure the reliability of the findings.

This review will have some limitations. Given the scarcity of studies evaluating psychological interventions targeted to the needs of caregivers of HSCT patients, a limited number of RCTs may be identified. This will limit our ability to conduct subgroup analysis, including treatment type (e.g., autologous vs. allogeneic), intervention approach (e.g., cognitive behavior therapy, mindfulness meditation), or modalities (e.g., in-person, digital). However, it is expected that enough research (i.e., > 5 studies) [31] has been conducted and published since the most recent review in 2018 (which included 4 RCTs), allowing for a quantitative synthesis using meta-analysis.

The findings of this review have the potential to expand the evidence in the field of psychological interventions addressing the needs of caregivers of HSCT patients. Additionally, the results will aid in identifying the characteristics of interventions that are most likely efficacious and inform future advancement of psychological interventions for caregivers.

## Supporting information

S1 Checklist PRISMA-P 2015 Checklist. (DOCX)

S2 Text Ovid MEDLINE (Wolters Kluwer) Search Strategy. (DOCX)

S3 Table Information to be extracted from eligible studies. (DOCX)

## Abbreviations

HSCT: Hematopoietic Stem Cell Transplant
QoL: Quality of Life
RCTs: Randomized Controlled Trials
PRISMA-P: Systematic Review and Meta-Analysis Protocols
PROSPERO: Prospective Register of Systematic Reviews Protocols
GRADE: Grading of Recommendations Assessment, Development, and Evaluation
AYAs: Adolescents and Young Adults
