# Supplementary material for: Effect of psychological interventions on outcomes for caregivers of hematopoietic stem cell transplant patients: Protocol for a systematic review and planned meta-analysis
Source: PLoS One. 2025 Aug 18;20(8):e0330323. doi: 10.1371/journal.pone.0330323 (PMC12360561; doi:10.1371/journal.pone.0330323)
Supplement: S2 Text — (DOCX) [file pone.0330323.s002.docx]

**S2 Text: Ovid MEDLINE (Wolters Kluwer) Search Strategy**

**Database:**
Ovid MEDLINE(R) ALL <1946 to February 16, 2024>

| **#** | **Query** | **Results from 19 Feb 2024** |
| --- | --- | --- |
| 1 | exp Allografts/ | 12,076 |
| 2 | Autografts/ | 4,216 |
| 3 | Bone Marrow Transplantation/ | 45,784 |
| 4 | exp Hematopoietic Stem Cell Transplantation/ | 58,633 |
| 5 | Transplantation, Autologous/ | 54,209 |
| 6 | Transplantation, Homologous/ | 86,505 |
| 7 | ((allogeneic or autologous or haematopoietic or haploidentical or haplo-identical or hematopoietic or marrow or peripheral or syngeneic) adj4 (graft* or transplant*)).mp. | 197,781 |
| 8 | ((allo?BMT or allo?HCST or allo?HCT or allo?HSCT or allo?SCT or auto?BMT or auto?HCST or auto?HCT or auto?HSCT or auto?SCT or BMT or haplo?BMT or haplo?HCST or haplo?HCT or haplo?HSCT or haplo?SCT or HCST or HCT or HSCT or SCT) adj4 (graft* or transplant*)).mp. | 33,590 |
| 9 | or/1-8 | 262,642 |
| 10 | Adult Children/ | 1,884 |
| 11 | Caregiver Burden/ | 699 |
| 12 | Caregivers/ | 52,147 |
| 13 | Family/ | 85,561 |
| 14 | Fathers/ | 11,198 |
| 15 | Friends/ | 7,012 |
| 16 | Grandparents/ | 771 |
| 17 | Mothers/ | 57,981 |
| 18 | Parent-Child Relations/ | 36,647 |
| 19 | Parents/ | 83,587 |
| 20 | Siblings/ | 13,574 |
| 21 | Single Parent/ | 1,245 |
| 22 | Spouses/ | 11,730 |
| 23 | (adult?child* or aunt* or boyfriend* or brother* or cousin* or daughter* or ex-husband* or ex-partner* or ex-spouse* or ex-wi#e* or friend* or girlfriend* or grandfather* or grandmother* or grandparent* or husband* or informal* or kin or mother* or neighbor* or nephew* or niece* or parent* or partner* or sibling* or sister* or son* or spouse* or uncle* or wi#e*).mp. | 3,717,603 |
| 24 | ((family or kinship or married) adj2 (member* or network* or person*)).mp. | 133,291 |
| 25 | or/10-24 | 3,882,904 |
| 26 | (carer* or care?giv* or care-giv* or care giv* or patient?caregiv*).mp. | 134,781 |
| 27 | 25 and 26 | 87,663 |
| 28 | exp Glioma/ | 101,705 |
| 29 | exp Hematologic Neoplasms/ | 25,762 |
| 30 | Hodgkin Disease/ | 36,055 |
| 31 | exp Leukemia/ | 257,123 |
| 32 | Leukemia, Lymphocytic, Chronic, B-Cell/ | 19,139 |
| 33 | exp Leukemia, Lymphoid/ | 83,014 |
| 34 | exp Leukemia, Myeloid/ | 108,879 |
| 35 | Leukemia, Myelomonocytic, Juvenile/ | 438 |
| 36 | Lymphoma/ | 54,830 |
| 37 | Lymphoma, Non-Hodgkin/ | 36,544 |
| 38 | Multiple Myeloma/ | 48,015 |
| 39 | Myelodysplastic Syndromes/ | 17,434 |
| 40 | Neoplasms/ | 514,497 |
| 41 | Neuroblastoma/ | 31,391 |
| 42 | exp Precursor Cell Lymphoblastic Leukemia-Lymphoma/ | 34,312 |
| 43 | Sarcoma, Ewing/ | 7,890 |
| 44 | ((acute or adult or ALL or B?Cell or childhood or chronic or cleaved?cell or CLL or CML or diffuse or disrupt* or familial or granlocyt* or granulom* or high?grade or Hodgkin$2 or intermediate?grade or JMML or juvenile or L1 or L2 or low?grade or lymphatic or lymphoblast* or lymphocyt* or lymphogranulom* or lymphoid or lymphoplasm* or mixed* or monoc* or myelo* or non?cleave* or non?Hodgkin* or Philadelphia?Positive or plasmacyt* or pleomorphic or Pre?B?Cell or small* or T?ALL or T?Cell or T?lympho* or undifferent* or Well?Different*) adj4 (leuk?emi* or lymphom* or malignan*)).mp. | 390,480 |
| 45 | (blood?cancer* or bone?marrow or cancer* or carcinom* or germinoblastom* or glioma* or h?ematolog* or h?ematop* or leucocyth?em* or lymphosarcom* or malignan* or metastat* or myeloma?multipl* or myelomatos* or neoplas* or neuroblastom* or reticulolymphosarcoma* or reticulosarcom*).mp. | 4,712,875 |
| 46 | ((dysmyelopoiet* or Ewing$2 or germinoblastic or glial or Kahler or lymphat* or MDS? or myleodysplas* or multiple or plasma?cell or reticulum?cell) adj4 (disease* or hematopoet* or myelom* or sarcom* or syndrome* or tumo?r*)).mp. | 190,192 |
| 47 | or/28-46 | 5,001,269 |
| 48 | or/9,47 | 5,138,614 |
| 49 | 27 and 48 | 8,620 |
| 50 | "Acceptance and Commitment Therapy"/ | 1,012 |
| 51 | Behavior Therapy/ | 30,758 |
| 52 | Cognitive Behavioral Therapy/ | 30,970 |
| 53 | Cognitive Restructuring/ | 30 |
| 54 | Counseling/ | 40,338 |
| 55 | Couples Therapy/ | 887 |
| 56 | Emotion-Focused Therapy/ | 74 |
| 57 | Family Therapy/ | 9,224 |
| 58 | Marital Therapy/ | 1,547 |
| 59 | Mindfulness/ | 6,696 |
| 60 | Mind-Body Therapies/ | 1,246 |
| 61 | Narrative Therapy/ | 256 |
| 62 | Palliative Care/ | 64,293 |
| 63 | Psychological Theory/ | 14,533 |
| 64 | Psychosocial Intervention/ | 1,093 |
| 65 | Psychotherapy/ | 58,407 |
| 66 | Psychotherapy, Group/ | 14,534 |
| 67 | Psycho-Oncology/ | 258 |
| 68 | Relaxation Therapy/ | 6,588 |
| 69 | Self Care/ | 36,378 |
| 70 | Self-Compassion/ | 359 |
| 71 | Self Efficacy/ | 24,758 |
| 72 | Social Support/ | 79,605 |
| 73 | Writing/ | 17,230 |
| 74 | (compassion focused or ecotherap* or oncopsycholog* or problem solving or psycho?oncolog* or self?awareness or self?care or self?compassion* or self?efficacy or self?forgiveness).mp. | 44,277 |
| 75 | ((acceptance or behavio?r* or BT or CBT or cognitive or commitment or community or conditioning or couple* or CT or emotion-focused or family or group or marital or marriage or MBTCT or mind?body or mindfulness or narrative or nature or palliative or process?experiential or psycho?social or relaxation or social or social?cognitive or social?environment*) adj3 (care or consult* or counsel* or interven* or learn* or mediat* or method* or modification* or oncolog* or psychiat* or psycholog* or psychotherap* or reframing* or restructur* or support* or techni* or theor* or therap* or training* or treatment*)).mp. | 1,118,783 |
| 76 | ((individual or personal) adj2 (correspond* or diary or journal* or poetry or workbook* or writing*)).mp. | 1,510 |
| 77 | or/50-76 | 1,290,510 |
| 78 | 49 and 77 | 4,771 |

exp Allografts/
Autografts/
Bone Marrow Transplantation/
exp Hematopoietic Stem Cell Transplantation/
Transplantation, Autologous/
Transplantation, Homologous/
((allogeneic or autologous or haematopoietic or haploidentical or haplo-identical or hematopoietic or marrow or peripheral or syngeneic) adj4 (graft* or transplant*)).mp.
((allo?BMT or allo?HCST or allo?HCT or allo?HSCT or allo?SCT or auto?BMT or auto?HCST or auto?HCT or auto?HSCT or auto?SCT or BMT or haplo?BMT or haplo?HCST or haplo?HCT or haplo?HSCT or haplo?SCT or HCST or HCT or HSCT or SCT) adj4 (graft* or transplant*)).mp.
or/1-8
Adult Children/
Caregiver Burden/
Caregivers/
Family/
Fathers/
Friends/
Grandparents/
Mothers/
Parent-Child Relations/
Parents/
Siblings/
Single Parent/
Spouses/
(adult?child* or aunt* or boyfriend* or brother* or cousin* or daughter* or ex-husband* or ex-partner* or ex-spouse* or ex-wi#e* or friend* or girlfriend* or grandfather* or grandmother* or grandparent* or husband* or informal* or kin or mother* or neighbor* or nephew* or niece* or parent* or partner* or sibling* or sister* or son* or spouse* or uncle* or wi#e*).mp.
((family or kinship or married) adj2 (member* or network* or person*)).mp.
or/10-24
(carer* or care?giv* or care-giv* or care giv* or patient?caregiv*).mp.
25 and 26
exp Glioma/
exp Hematologic Neoplasms/
Hodgkin Disease/
exp Leukemia/
Leukemia, Lymphocytic, Chronic, B-Cell/
exp Leukemia, Lymphoid/
exp Leukemia, Myeloid/
Leukemia, Myelomonocytic, Juvenile/
Lymphoma/
Lymphoma, Non-Hodgkin/
Multiple Myeloma/
Myelodysplastic Syndromes/
Neoplasms/
Neuroblastoma/
exp Precursor Cell Lymphoblastic Leukemia-Lymphoma/
Sarcoma, Ewing/
((acute or adult or ALL or B?Cell or childhood or chronic or cleaved?cell or CLL or CML or diffuse or disrupt* or familial or granlocyt* or granulom* or high?grade or Hodgkin$2 or intermediate?grade or JMML or juvenile or L1 or L2 or low?grade or lymphatic or lymphoblast* or lymphocyt* or lymphogranulom* or lymphoid or lymphoplasm* or mixed* or monoc* or myelo* or non?cleave* or non?Hodgkin* or Philadelphia?Positive or plasmacyt* or pleomorphic or Pre?B?Cell or small* or T?ALL or T?Cell or T?lympho* or undifferent* or Well?Different*) adj4 (leuk?emi* or lymphom* or malignan*)).mp.
(blood?cancer* or bone?marrow or cancer* or carcinom* or germinoblastom* or glioma* or h?ematolog* or h?ematop* or leucocyth?em* or lymphosarcom* or malignan* or metastat* or myeloma?multipl* or myelomatos* or neoplas* or neuroblastom* or reticulolymphosarcoma* or reticulosarcom*).mp.
((dysmyelopoiet* or Ewing$2 or germinoblastic or glial or Kahler or lymphat* or MDS? or myleodysplas* or multiple or plasma?cell or reticulum?cell) adj4 (disease* or hematopoet* or myelom* or sarcom* or syndrome* or tumo?r*)).mp.
or/28-46
or/9,47
27 and 48
"Acceptance and Commitment Therapy"/
Behavior Therapy/
Cognitive Behavioral Therapy/
Cognitive Restructuring/
Counseling/
Couples Therapy/
Emotion-Focused Therapy/
Family Therapy/
Marital Therapy/
Mindfulness/
Mind-Body Therapies/
Narrative Therapy/
Palliative Care/
Psychological Theory/
Psychosocial Intervention/
Psychotherapy/
Psychotherapy, Group/
Psycho-Oncology/
Relaxation Therapy/
Self Care/
Self-Compassion/
Self Efficacy/
Social Support/
Writing/
(compassion focused or ecotherap* or oncopsycholog* or problem solving or psycho?oncolog* or self?awareness or self?care or self?compassion* or self?efficacy or self?forgiveness).mp.
((acceptance or behavio?r* or BT or CBT or cognitive or commitment or community or conditioning or couple* or CT or emotion-focused or family or group or marital or marriage or MBTCT or mind?body or mindfulness or narrative or nature or palliative or process?experiential or psycho?social or relaxation or social or social?cognitive or social?environment*) adj3 (care or consult* or counsel* or interven* or learn* or mediat* or method* or modification* or oncolog* or psychiat* or psycholog* or psychotherap* or reframing* or restructur* or support* or techni* or theor* or therap* or training* or treatment*)).mp.
((individual or personal) adj2 (correspond* or diary or journal* or poetry or workbook* or writing*)).mp.
or/50-76
49 and 77
