# Supplementary material for: Effect of psychological interventions on outcomes for caregivers of hematopoietic stem cell transplant patients: Protocol for a systematic review and planned meta-analysis
Source: PLoS One. 2025 Aug 18;20(8):e0330323. doi: 10.1371/journal.pone.0330323 (PMC12360561; doi:10.1371/journal.pone.0330323)
Supplement: S3 Table — (DOCX) [file pone.0330323.s003.docx]

**S3 Table. Information to be extracted from eligible studies**

| **Information to be extracted** | **Data item examples** |
| --- | --- |
| Publication details | First author, year, type, Journal |
| **Study** |  |
| General characteristics | Study design, duration of study and follow-up, location, description of comparison/control group |
| Population/participants | Target population (e.g., autologous or allogeneic), inclusion and exclusion criteria, demographic characteristics (e.g., age, gender, race, ethnicity, income, employment status, relationship to patient), sample size of intervention and comparison groups at each follow-up of interest |
| Procedures | Recruitment strategies, randomization (e.g., randomized control trial, cohort study), method to handle missing data, study setting (e.g., clinical, community-based) |
| Intervention Characteristics | Type of intervention (e.g., cognitive behavioral therapy, mindfulness), delivery modality (in-person, mobile app, web-based), language availability, the use of theory, formative research, setting, duration/intensity, characteristics of control intervention if available |
| Feasibility and Acceptability | Feasibility (e.g., recruitment, retention, and adherence), acceptability measures |
| Outcomes/Results (for intervention and control conditions) | Sample size, Means and SDs:  1. Self-reported mental health outcomes (e.g., burden, distress, anxiety, depression),  2. Physiological outcomes (e.g., immunological markers, metabolic markers)  3. Patient-reported outcomes (e.g., depression, stress, healthcare utilization) |
| Miscellaneous | Limitations, funding sources, ethical approval, references, and key conclusion |
